# Temporal dynamics of seed excretion by wild ungulates: implications for plant dispersal

**DOI:** 10.1002/ece3.1512

**Published:** 2015-06-06

**Authors:** Mélanie Picard, Julien Papaïx, Frédéric Gosselin, Denis Picot, Eric Bideau, Christophe Baltzinger

**Affiliations:** 1 Irstea, UR EFNO, centre de Nogent-sur-VernissonF-45290, Nogent-sur-Vernisson, France; 2 UR BIOGER, INRA, Avenue Lucien BrétignièresBP01, 78850, Thiverval-Grignon, France; 3 UR MIAJ, INRA, Domaine de Vilvert78352, Jouy-en-Josas CEDEX, France; 4 Centre d’Ecologie Fonctionnelle et Evolutive, UMR 5175, campus CNRS1919 Route de Mende, 34293, Montpellier 5, France; 5 UR CEFS, INRABP 52627, 31326, Castanet-Tolosan, France

**Keywords:** Bayesian dynamic model, digestion mode, endozoochory, metacommunity dynamics, retention time, ruminants

## Abstract

Dispersal is a key process in metapopulation dynamics as it conditions species’ spatial responses to gradients of abiotic and biotic conditions and triggers individual and gene flows. In the numerous plants that are dispersed through seed consumption by herbivores (endozoochory), the distance and effectiveness of dispersal is determined by the combined effects of seed retention time in the vector’s digestive system, the spatial extent of its movements, and the ability of the seeds to germinate once released. Estimating these three parameters from experimental data is therefore crucial to calibrate mechanistic metacommunity models of plant–herbivore interactions. In this study, we jointly estimated the retention time and germination probability of six herbaceous plants transported by roe deer (*Capreolus capreolus*), red deer (*Cervus elaphus*), and wild boar (*Sus scrofa*) through feeding experiments and a Bayesian dynamic model. Retention time was longer in the nonruminant wild boar (>36 h) than in the two ruminant species (roe deer: 18–36 h, red deer: 3–36 h). In the two ruminants, but not in wild boar, small and round seeds were excreted faster than large ones. Low germination probabilities of the excreted seeds reflected the high cost imposed by endozoochory on plant survival. Trait-mediated variations in retention time and germination probability among animal and plant species may impact plant dispersal distances and interact with biotic and abiotic conditions at the release site to shape the spatial patterns of dispersed plant species.

## Introduction

Habitat loss and fragmentation may disrupt metapopulation dynamics by isolating populations in space, thus impairing local and regional species persistence (Cain et al. 2000; Fahrig 2003; Soons et al. 2005). In island-based formulations of the metapopulation theory, connectivity among distant populations mainly depends on spatial patterns of suitable habitat patches and on species’ ability to reach these habitats through unsuitable matrices (Soons et al. 2005). Quantifying dispersal processes is therefore crucial to explain spatial patterns of species occurrence and abundance dynamics, as well as community assembly processes.

In zoochory, dispersal distances are the product of the probability of encroachment or consumption of a seed on/by its vector, the duration of the seed retention by the vector (retention time), and the distance covered by the vector during this time. These three parameters are influenced by the ecological characteristics of both the transported plants and the vector. In the case of endozoochory (dispersal following plant consumption by an animal), fruit releasing height, its color, and animal habitat and feeding preferences influence probability of being swallowed. In herbaceous plant species, foliage attracts the vector with its palatability and high nutrient contents, thus acting as a fruit (Janzen 1984). Digestion time mainly depends on animal physiology and food quality (Holand 1994; Jiang and Hudson 1996; Elston and Hewitt 2010), while the distance covered by the vector is influenced by its habitat preferences, the extant and habitat composition of its home range, and the landscape configuration.

Endozoochory has been studied for a diversity of vectors including birds (Murray 1988), bats (Muscarella and Fleming 2007), rodents (Forget and Milleron 1991), and large mammals (Malo and Suárez 1995a,b; Couvreur et al. 2005; Eycott et al. 2007; Jaroszewicz et al. 2013). Among these, species with large home range and large intake capacities, including ungulates, are potentially effective vectors for long-distance dispersal in herbaceous plants (Will and Tackenberg 2008). While ungulate movements and the distances they cover are easily accessible through GPS monitoring, experimental assessments of retention time remain rare because they rely on individual monitoring in controlled conditions with heavy logistic constraints and associated small sample sizes. Endozoochorous seed retention time has rarely been quantified, but mostly for livestock (sheep, cattle, horse, and donkey, by Cosyns et al. 2005), with the exception of single studies on fallow deer (Mouissie et al. 2005) and moose (Seefeldt et al. 2010). As an illustration of the weaknesses of existing data, Will and Tackenberg (2008) calibrated a model of endozoochorous plant dispersal based on a single study with data from feeding experiments involving sheep and cattle (Bonn 2005), while D’hondt et al. (2012) assumed a theoretical retention-time distribution in order to model endozoochory by cattle. Furthermore, the parameters of endozoochory mediated by wild ungulates and livestock may differ due to differences in species’ feeding preferences, movements, and digestion strategy. Hence, calibrating dispersal model with experiments on livestock may not reflect the actual dynamics of endozoochory in plant communities that primarily interact with wild ungulates.

Food retention time increases with the vector’s body mass (Illius and Gordon 1992), but also depends on its digestion mode (ruminant or not, Elston and Hewitt 2010) and feeding preferences (browser or grazer). In ruminants, the fine fraction of ingested plants (including small seeds) is passed onwards in the digestive tract, while larger particles (including large seeds) are selectively retained to be chewed twice and further broken down (Clauss et al. 2009). Schwarm et al. (2008) showed that in nonruminants, this particle sorting mechanism is reversed. Thus, retention time probably differs as a function of seed size and the vector’s digestion mode. Furthermore, among ruminants, seed retention time is expected to be longer in grazers (grass and roughage eaters) than in browsers (concentrate selectors feeding on forbs, shrub, leaves, and stems) due to the low digestibility of fibers present in high quantities in grasses (Hofmann 1989; Behrend et al. 2004). Mixed feeders like red deer are either browser or grazer according to seasonal vegetation availability, which implies that their influence as dispersal vectors changes with time. Consistent variations in retention time according to food quality were observed in red deer and roe deer (Holand 1994; Jiang and Hudson 1996).

Dispersal is only effective if a sufficient number of seeds germinate once released (Schupp et al. 2010), but endozoochory imposes high costs on seeds due to mastication and exposure to digestive enzymes, such that the survival percentage of defecated seeds is low (Cosyns et al. 2005; Mouissie et al. 2005). Seed morphology influences seed survival after their passage through the digestive system. Seeds that germinate after defecation by ruminant species often share a suite of physical characteristics that enhance germination probability, including a small size, round shape, low mass, and a hard seed coat (Pakeman et al. 2002; Couvreur et al. 2005; Mouissie et al. 2005), although smaller proportions of virtually any seeds have been found in animal feces. Pakeman et al. (2002) suggested that the characteristics that allow seed survival in a soil seedbank, including small size and round seeds (Thompson et al. 1993), may also enhance seed survival after excretion. Furthermore, small and round seeds probably have shorter retention times (Lauper et al. 2013) that limit their exposure to digestive enzymes and may thus increase postrelease survival probability. However, these trait–germination relationships have received mixed support. For instance, Mouissie et al. (2005) found no relation between mean seed retention time and seed shape, mass, and longevity, while D’hondt and Hoffmann (2011) failed to explain postrelease seed mortality with seed size and shape. They proposed instead that seed coat impermeability to water (i.e., physical dormancy) could increase seed survival. The ecological significance of endozoochory for plant dispersal therefore probably differs among seeds with differing morphologies and compositions, although this remains to be experimentally tested.

Using a comparative experimental approach, we quantified endozoochorous seed retention times and germination rates for six plant species abundant in western Europe and frequently consumed by wild ungulates (*Calluna vulgaris* L., *Juncus effusus* L., *Plantago media* L., *Prunella vulgaris* L., *Rubus fruticosus* L., and *Trifolium pratense* L.). We tested differences in retention times among three vectors: roe deer (*Capreolus capreolus*: a small browser ruminant), red deer (*Cervus elaphus*: a large intermediate mixed-feeder ruminant), and wild boar (*Sus scrofa*: an omnivore–frugivore nonruminant) (Hofmann 1989; Clauss et al. 2008). These three vectors are assumed to differ in their retention times because of their differences in digestion modes, feeding preferences, and body masses. Contrary to domesticated ungulates, they occupy large home ranges not limited by human confinement and should as a result disperse seeds over longer distances. However, data on seed retention time by these wild animals are still needed, to calibrate mechanistic models on seed dispersal. We tested four predictions:

1. Seed retention time is shorter in a small-bodied browser ruminant (roe deer) than in a larger mixed-feeder ruminant (red deer).
2. For the roe and red deer ruminants, median retention time is shorter for plant species bearing small, round, light, and/or long-lived seeds with a hard coat than for plant species with opposite traits.
3. For the nonruminant wild boar, the reverse pattern should be found: plant species with small seeds (*Juncus effusus* and *Calluna vulgaris*) should have longer median retention times than large seeds.
4. A shorter median retention time should increase median germination probability.

From (2) to (4), we thus expected higher germination rates for *Juncus effusus* and *Calluna vulgaris*, whose seeds share all the characteristics associated with short retention times in (2) than for other seeds for the two ruminant species, and the reverse trend for wild boar.

## Materials and Methods

### Feeding experiments

**Figure 1:**
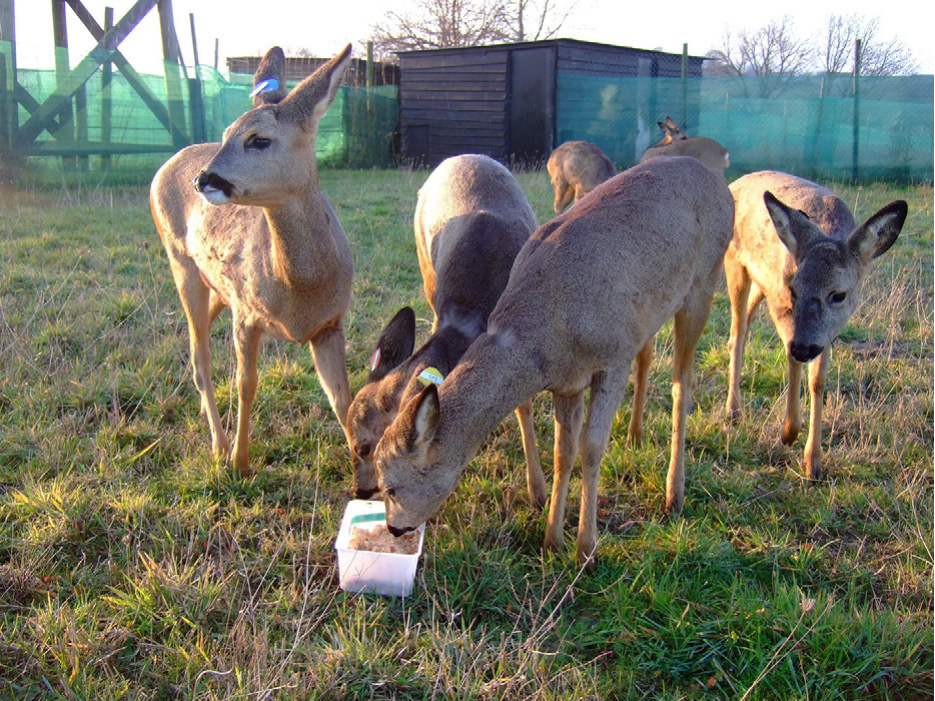
Captive roe deer (*Capreolus capreolus*) used in the feeding experiments, in Gardouch, France. Photo © Mélanie Picard.

We conducted feeding experiments on captive animals in three experimental platforms used to work with wild animals (see more details in Appendix S1). All experiments complied with the ethical standards of animal manipulation as defined by the French laws on animal welfare (Décret n°2013-118, see Appendix S1 for the licenses numbers). We monitored eleven individual animals of the three species (Fig.1): five young roe deer (four females and one male), four young female red deer, and two adult wild boars (one female and one male). Body mass per species, respectively, averaged 21.2 ± 4.6 kg, 53.3 ± 5.2 kg, and 100 kg (hereafter ± standard deviation unless otherwise specified). Retention time can vary between sexes due to sexual size dimorphism, but this was not the case in our experiment, as both sexes weighed roughly the same in roe deer and wild boar. We replicated the monitoring six times for each animal species; some individuals were thus used in several replicates.

Estimating retention time critically requires that the initiation of the individual monitoring corresponds precisely to the time at which all the seeds are ingested. We therefore adapted seed quantities to the intake capacity of our animal vectors (Table1), which is lower than that of livestock (in particular, roe deer cannot ingest large seed quantities over a short time lag). We limited our study to six plant species, to ensure sufficient seed sample sizes, and mixed controlled seed quantities to constitute seed mixtures in which seed proportions reflected the relative natural seed production of each plant species (Table1). We measured several seed traits in the initial pure seed samples from commercial suppliers to serve as a quantitative support to the interpretation of our results (see Table1).

**Table 1:** Plant species used in the feeding experiments and their corresponding seed characteristics

Plant species	Seed size (mm)	Seed mass (mg)	Seed shape	Seed longevity index	Seed number per flower//plant	Seed quantity in a seed mixture	
						Roe deer	Red deer and Wild boar
*Calluna vulgaris* L.	0.5 × 0.4 (small)	0.05 ± 0.04 (light)	0.03 (round)	0.81	10–100//>10,000	11,000	15,000
*Juncus effusus* L.	0.5 × 0.2 (small)	0.01 ± 0.01 (light)	0.08 (rather round)	0.93	10–100//>10,000	11,000	15000
*Plantago media* L.	2.0 × 1.0 (large)	0.44 ± 0.01 (medium)	0.14 (rather flat or elongated)	0.29	<10//1000–10,000	1500	2000
*Prunella vulgaris* L.	1.9 × 1.1 (large)	0.73 ± 0.06 (medium)	0.05 (rather round)	0.20	<10//100–1000	1500	2000
*Rubus fruticosus* L.	3.0 × 2.0 (large)	3.53 ± 0.08 (heavy)	0.07 (rather round)	0.11	10–100//NA	1500	2000
*Trifolium pratense* L.	2.0 × 1.4 (large)	1.51 ± 0.01 (heavy)	0.04 (round)	0.24	<10//NA	1500	2000

Seed size is expressed as length × width (in millimeters, measured in a random sample of 50 seeds per species). Mean mass ± SD (in milligrams) was measured in a random sample of 100 air-dried seeds per species. Seed shape corresponds to variance in dimensions *Vs*, calculated following Bekker et al. (1998), ranking from 0: perfectly spherical, to 0.2: flat or elongated. Seed longevity index ranks from 0: no persistent records, to 1: all records persistent in Thompson et al.’s database (1997) or in the LEDA Traitbase (Kleyer et al. 2008) for *Rubus fruticosus*. Seed numbers are from Ecoflora database (Fitter and Peat 1994). NA indicates “no data.”

Feeding experiments were conducted from June 2009 to November 2010. Each animal was isolated in a cleaned enclosure (roe deer and wild boar) or box (red deer). Each individual was then fed with a seed mixture mixed with its usual food in a bucket to facilitate ingestion: granules (roe deer), triticale (mixed with hay during the experiment; red deer), and pears (wild boar). As the animals were fed with their usual food, no adaptation period was needed before the experiments. Prior to feeding animals, we checked that seeds present in the hay were absent from the seed mixtures. Additionally, all feces found in the enclosures and boxes were removed before the onset of the monitoring and kept to serve as controls for possible seed contamination. We began the experiments in the morning, ensuring that each animal ingested as many seeds as possible. We rubbed the animal muzzle above the bucket and added food so that it swallowed seeds while eating anew. We interrupted the feeding phase whether all seeds appeared to be ingested or the animal refused to eat. It took as long as 20 min for roe deer. We fixed *t* = 0 as the time of the last ingestion. After the feeding phase, we collected unconsumed seeds that remained in buckets and counted them under stereomicroscope to estimate the percentage of ingested seeds by every animal, which ranged from 87.3% to 100% (mean = 95.8 ± 3.0%). Note that some seeds may have fallen unnoticed on the soil during the experiment, in quantities likely too small to affect the results of our experiment. During the experiments, the animals had free access to freshwater and received their usual food every day.

We used Illius and Gordon’s equations (1992) for ruminants and hindgut fermenters relating mean retention time to animal body mass to determine the maximum duration of individual monitoring necessary according to the body mass values of our animal species. For ruminants, we predicted a longer mean retention time (42.8 h) for red deer, with a maximum weight of 60 kg, than for roe deer (34.3 h for 25 kg), while mean retention time should be lower for the hindgut fermenter wild boar (30.4 h for 100 kg). To ensure that we covered the estimated maximum retention duration, we collected all fresh feces for 54 h, from seed ingestion (*t* = 0), every 3 h during the first 24 h and every 6 h thereafter.

### Seed release and germination

Dissecting entirely all the collected feces for seed extraction revealed intractable. Hence, we extracted and dissected two random samples of 4.0 g for roe deer or 8.0 g for red deer and wild boar from each feces. Sampled weight reflected the average feces weight of each species, which was lower for roe deer (24.1 ± 17.7 g) than for wild boar (42.6 ± 23.2 g) and red deer (61.9 ± 32.9 g). We dissected the first sample (“dissected sample”) under stereomicroscope, to visually identify and count the seeds that passed animal guts. We used the second sample (“germination sample”) to assess postrelease seed germination after a 1-month vernalization period in a cold chamber (4°C). We washed each germination sample through sieves of 2 mm, 800 *μ*m, 400 *μ*m, and 200 *μ*m stacked on top of each other, which, respectively, retained large components, large seeds (*Plantago media*, *Prunella vulgaris*, *Trifolium pratense,* and *Rubus fruticosus*), medium components, and small seeds (*Calluna vulgaris* and *Juncus effusus*). This process also removed fungi spores. We placed the contents of the 800 *μ*m and 200 *μ*m sieves together on wet blotting paper as a 3-mm-thick layer in germination boxes. For each plant species, we also prepared a control box equally divided in four replicates of 100 noningested seeds. We monitored all the germination boxes under controlled conditions in a growth chamber, with daily cycles of 16 h of light at 25°C and 8 h of darkness at 15°C, and water supply when necessary. These conditions are supposed to allow germination of a large range of plant species. We counted and identified seedlings twice a week for 2 months. We thus obtained standardized seed and seedling counts for each dissected and germination sample, for a total of 154 feces (roe deer: 53, red deer: 64, and wild boar: 37 feces). We calculated the germination percentage for the noningested seeds by averaging the number of seedlings in each of the four replicates of 100 noningested seeds.

### Statistical analyses

**Figure 2:**
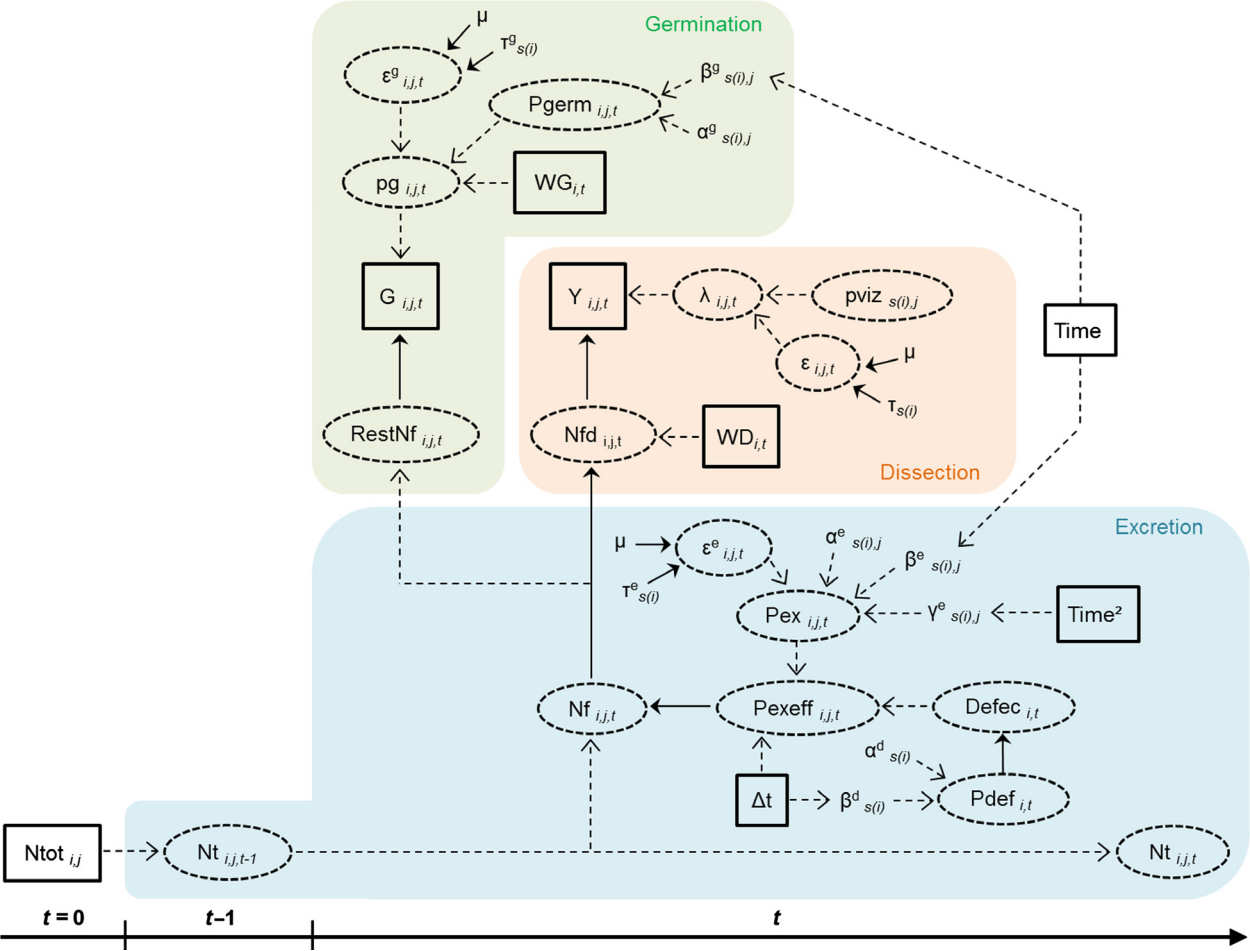
Schematic representation of the Bayesian model. Subscripts correspond to individual *i*, animal species *s(i)*, plant species *j,* and time *t*. Δ*t* is the time lag between *t* and *t−1*. The notations for the different submodels are as follow. Excretion submodel: observed number of seeds ingested (*Ntot*_*i,j*_), number of seeds in the digestive system at *t−1* (*Nt*_*i,j,t−1*_) and *t* (*Nt*_*i,j,t*_), number of seeds excreted in a whole feces (*Nf*_*i,j,t*_, with *Nt*_*i,j,t*_ = *Nt*_*i,j,t-*1_ − *Nf*_*i,j,t*_), excretion probability (*pexeff*_*i,j,t*_), defecation occurrence (0 or 1: *defec*_*i,t*_), potential excretion probability (*pex*_*i,j,t*_), and defecation probability (*pdef*_*i,t*_). Dissection submodel: number of seeds in the dissected sample (*Nfd*_*i,j,t*_), relative weight of the dissected sample (*WD*_*i,t*_), seeds counted in the dissected sample (*Y*_*i,j,t*_), probability of being counted (*λ*_*i,j,t*_), and seed detection probability (*pviz*_*s(i),j*_). Germination submodel: number of seeds in the nondissected part of a feces (*restNf*_*i,j,*t_), seedlings counted in the germination sample (*G*_*i,j,t*_), relative weight of the germination sample (WG_*i,t*_), probability of occurrence in the germination sample (*pg*_*i,j,t*_), and germination probability (*pgerm*_*i,j,t*_). *ε*_*i,j,t*_, 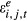, and 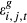 are random effects. 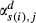, 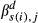, 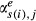, 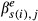, 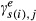
*,* 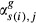, 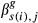 are the different probability distribution parameters. Solid squares and dashed circles, respectively, represent observed and latent variables. Solid and dashed arrows, respectively, represent stochastic and deterministic relationships. The detailed relationships among these variables can be found in the text.

We modeled the dynamics of seed excretion and seed germination together, which, instead of all previous similar studies, allowed us to estimate jointly retention time and its cost on seed viability. A Bayesian state/space formulation allowed us to represent both the observation process through observation variables (seed and seedling counts) and the system process through unobserved latent variables. The model is composed of three submodels (Fig.2). The excretion submodel describes the process of defecation and seed excretion, the dissection submodel describes the seed count experiment, and the germination submodel describes the germination of excreted seeds in the germination sample.

#### Excretion submodel

At the beginning of the experiment (*t* = 0), the number of ingested seeds of plant species *j* by animal *i* (*Nt*_*i,j,0*_) was modeled as a Poisson distribution with mean *Ntot*_*i,j*_:

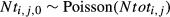


*Ntot*_*i,j*_ corresponds to the observed number of seeds of plant *j* ingested by animal *i*. After seed ingestion, the number of seeds in the digestive system at a given *t* (*Nt*_*i,j,t*_) is given by:

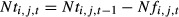


where *Nf*_*i,j,t*_ is the number of seeds that are excreted during the time interval between *t* and *t-1*. *Nf*_*i,j,t*_ is drawn from *Nt*_*i,j,t-1*_ in a binomial distribution with a probability *pexeff*_*i,j,t*_ (excretion probability):

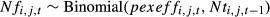


The excretion probability was defined as *pexeff*_*i,j,t*_ = *defec*_*i,t*_* *× *pex*_*i,j,t*_ × Δ_*t*_. The binary variable *defec*_*i,t*_ represents the defecation by animal *i* at time *t* (1 for excretion, 0 otherwise), modeled as a Bernoulli distribution with probability *pdef*_*i,t*_ (defecation probability):

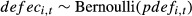


The defecation probability was then related to the time lag between two observation times (Δ*t*) and was allowed to vary among animal species *s(i)*:

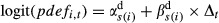


The potential excretion probability per time step *pex*_*i,j,t*_ was related through a logit link to a quadratic function of time (which permits *pex*_*i,j,t*_ to have a maximum) and was allowed to vary among animal species *s(i)* and plant species *j*:

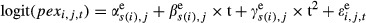


where 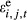 is a normally distributed random effect, in which variance *τ*^e^ varied among animal species:

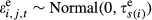


#### Dissection submodel

The actual number of seeds in the dissected sample (*Nfd*_*i,j,t*_) was sampled in the total number of seeds in the whole feces (*Nf*_*i,j,*t_) proportionally to the weight of the dissected sample (*Wd*_*i,t*_) relative to the weight of the whole feces (*W*_*i,t*_), *WD*_*i,t*_ = *Wd*_*i,t*_/*W*_*i,t*_, as a binomial distribution:

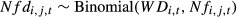


The number of seeds counted in the dissected sample (*Y*_*i,j,t*_) was then drawn from *Nfd*_*i,j,t*_ in a binomial distribution with a probability *λ*_*i,j,t*_, representing imperfect seed detection:

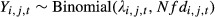


*λ*_*i,j,t*_ was related to seed detection probability (*pviz*_*s(i),j*_), with a normally distributed random effect (*ε*_*i,j,t*_ ∼ Normal (0,*τ*_*s(i)*_)):

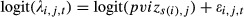


#### Germination submodel

The number of seedlings counted in the germination sample (*G*_*i,j,t*_) was a subsample of the number of seeds in the nondissected part of the feces (*restNf*_*i,j,t*_ = *Nf*_*i,j,t*_ − *Nfd*_*i,j,*t_) and depended on the relative weight of the germination sample: WG_*i,t*_ = (*Wg*_*i,t*_ / *W*_*i,t*_) / (1 − *WD*_*i,t*_), where *Wg*_*i,t*_ is the absolute weight of the germination sample. Thus, *G*_*i,j,t*_ was modeled so as to be drawn from *restNf*_*i,j,t*_ in a binomial distribution with a probability *pg*_*i,j,t*_:

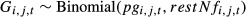


pg_i*,t*_ was related through a logit link function to germination probability (*pgerm*_*i,j,t*_) for the given relative weight of the germination sample (WG_*i,t*_) and to a normally distributed random effect 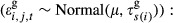


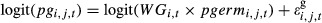


*pgerm*_*i,j,t*_ was linearly related to time, with variations among animal and plant species:

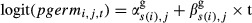


#### Run

Noninformative priors were specified for all parameters (Kéry 2010). Three Monte Carlo Markov chains (MCMC) were run under JAGS 3.3.0 (Plummer 2003), on one million iterations of burn-in and an additional million iterations, thinned by 500.

### Analyses

Chain mixing and convergence were assessed with the Gelman and Rubin statistic (Rhat) and were considered acceptable when Rhat < 1.2 (Gelman et al. 2004). Model fit was assessed through a posterior predictive check based on the posterior distributions of replicated data (*Y.rep*_*i,j,t*_ and *G.rep*_*i,j,t*_) (Gelman et al. 2004). The posterior distributions of the replicated data showed an adequate fit (see Appendix S2).

We calculated the mean percentage of seeds excreted during 54 h by each animal species. We then compared defecation, excretion, and germination probabilities between animal and plant species by computing the percentage of overlap in their posterior distributions, for each plant pair and animal species pair. We further computed median defecation probability (*pdef*_*i,t*_) and its associated 95% credibility interval for each animal species and the median maximal *pgerm*_*i,j,t*_ for each plant–animal species pair. We compared retention times across plant–animal species pairs by calculating the time associated with the maximum excretion probability (*pex*_*i,j,t*_).

## Results

### Experimental results

No seeds of the experimental plant species were found in any of the feces collected from the enclosures and boxes just before seed ingestion, indicating that our experiment was not polluted by the intrusion of external seeds. Roe deer, red deer, and wild boar, respectively, defecated 6.3 ± 1.5, 5.4 ± 1.4, and 4.0 ± 1.4 feces per day, on average 152.8 ± 62.7, 334.4 ± 87.9, and 172.4 ± 65.5 g of feces per day.

### Bayesian model results

#### Defecation

**Figure 3:**
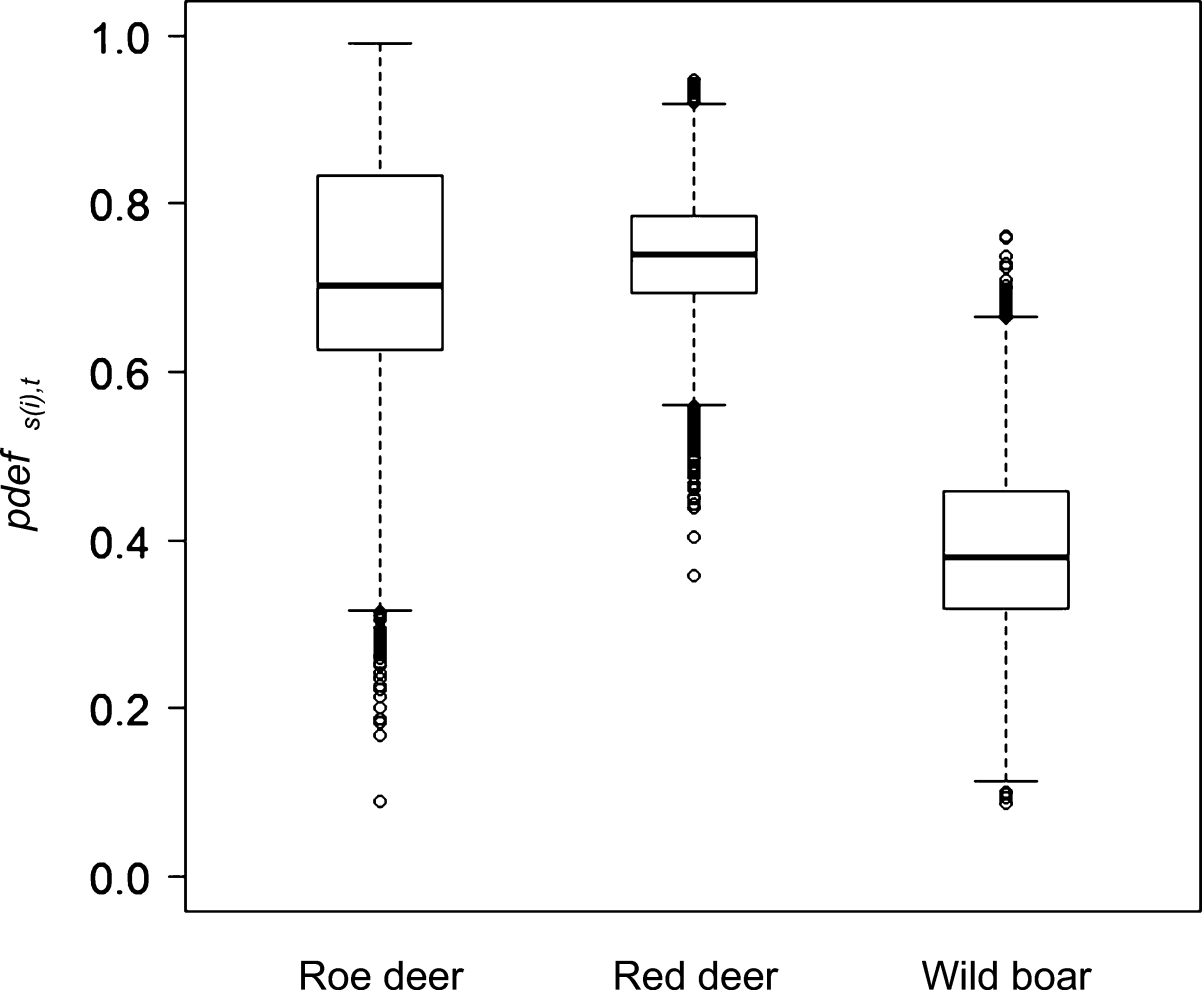
Defecation probability. Defecation probability (*pdef*_*s(i),t*_) by roe deer, red deer, and wild boar, from left to right. Boxplots show the median, 25th and 75th percentile.

Defecation probability (*pdef*_*s(i),t*_) was lower in wild boar (0.38 [0.21; 0.61]; hereafter median [2.5; 97.5% quantiles] unless otherwise specified) than in roe deer (0.70 [0.42; 0.94]) and red deer (0.74 [0.59; 0.87], posterior probability that *pdef*_*i,t*__wild boar_ < *pdef*_*i,t*__ruminants_ was 99.5 ± 0.3%), and similar in roe deer and red deer (*pdef*_*i,t*__red deer_ > *pdef*_*i,t*__roe deer_: 55.3%, Fig.3).

#### Seed excretion and retention time

**Figure 4:**
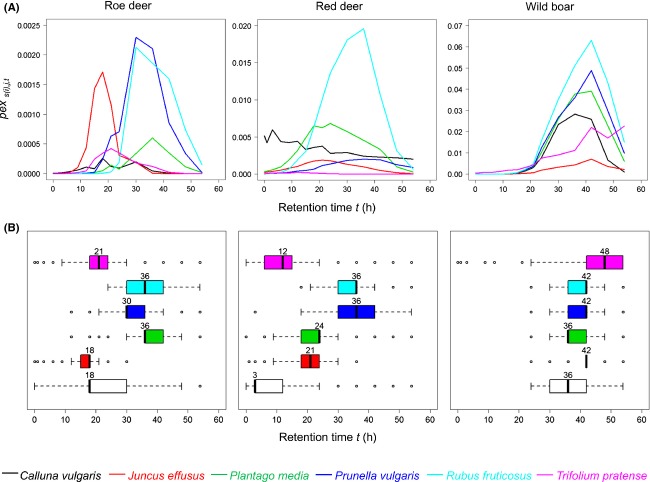
Excretion probability and time associated with maximum excretion probability. (A) Median excretion probability (*pex*_*s(i),j,t*_) through time, represented with a different scale for each animal species and without its credibility intervals (which were large) for better readability. (B) Time associated with the maximum *pex*_*s(i),j,t*_ (boxplots with median, 25th and 75th percentile).

Roe deer, red deer, and wild boar excreted, respectively, 3.6 ± 4.9, 7.8 ± 4.9, and 34.7 ± 17.2% of the seeds ingested. As a result, excretion probability was higher in wild boar than in the two ruminant species (posterior probability that *pex*_*i,j,t*__wild boar_ > *pex*_*i,j,t*__ruminants_ was 98.7 ± 1.8% for all plant species, Fig.4A). Median retention times (MRT) did not differ between the two ruminant species, although red deer exhibited a wider range of MRT than roe deer (roe deer: 25.5 ± 7.8 h, red deer: 22.5 ± 11.9 h, Table2). Wild boar had a longer retention time, irrespective to the plant species (42 ± 4.1 h). In roe deer, seeds of *Juncus effusus*, *Calluna vulgaris,* and *Trifolium pratense* were excreted faster than other seeds (Fig.4B and Table2). In red deer, we observed roughly the same pattern. The seeds of *Trifolium pratense* and *Calluna vulgaris* were excreted first (posterior probability that MRT _Trifolium pratense, Calluna vulgaris_ <* *MRT _other species_ was > 82.4%), followed by *Juncus effusus* and *Plantago media* (posterior probability that MRT_Juncus effusus, Plantago media_ <* *MRT _Rubus fruticosus, Prunella vulgaris_ was >83.0%) and then *Rubus fruticosus* and *Prunella vulgaris*. In wild boar, retention times were globally homogenous, although *Juncus effusus* and *Trifolium pratense* tended to be excreted later than the other species (posterior probability that MRT_Juncus effusus_ > MRT _other four species_ was >63.2% and that MRT_*Trifolium pratense*_ > MRT _other four species_ was >61.2%). Nevertheless, in wild boar, the excretion probability of *Juncus effusus* had only begun to decrease 54 h after ingestion and it was still increasing for *Trifolium pratense*, suggesting that seed release continued after the experiment (Fig.4A).

**Table 2:** Maximum excretion probability and its associated retention time

Plant species		Calluna vulgaris	Juncus effusus	Plantago media	Prunella vulgaris	Rubus fruticosus	Trifolium pratense
Roe deer	*Max pex*_*s(i),j,t*_	0.0004 [1^E^-4; 0.005]	0.0023 [9^E^-4; 0.014]	0.0009 [1^E^-4; 0.009]	0.0032 [9^E^-4; 0.017]	0.0034 [0.001; 0.019]	0.0008 [1^E^-4; 0.007]
	MRT	18 h [1; 36 h]	18 h [12; 24 h]	36 h [21; 54 h]	30 h [21; 42 h]	36 h [30; 54 h]	21 h [15; 36 h]
Red deer	*Max pex*_*s(i),j,t*_	0.0075 [0.003; 0.026]	0.0022 [0.001; 0.004]	0.0086 [0.004; 0.026]	0.0026 [0.001; 0.007]	0.0223 [0.012; 0.052]	0.0003 [1^E^-4; 7^E^-4]
	MRT	3 h [0; 54 h]	21 h [15; 30 h]	24 h [15; 36 h]	36 h [24; 54 h]	36 h [24; 42 h]	12 h [0; 21 h]
Wild boar	*Max pex*_*s(i),j,t*_	0.0360 [0.013; 0.103]	0.0083 [0.004; 0.041]	0.0472 [0.026; 0.104]	0.0536 [0.033; 0.100]	0.0714 [0.045; 0.148]	0.0331 [0.013; 0.202]
	MRT	36 h [30; 42 h]	42 h [30; 54 h]	36 h [30; 48 h]	42 h [30; 54 h]	42 h [30; 54 h]	48 h [30; 54 h]

Median maximum excretion probability (*Max pex*_*s(i),j,t*_) and its associated median retention time (MRT), with their 95% credibility interval [2.5; 97.5% quantiles].

#### Germination

**Figure 5:**
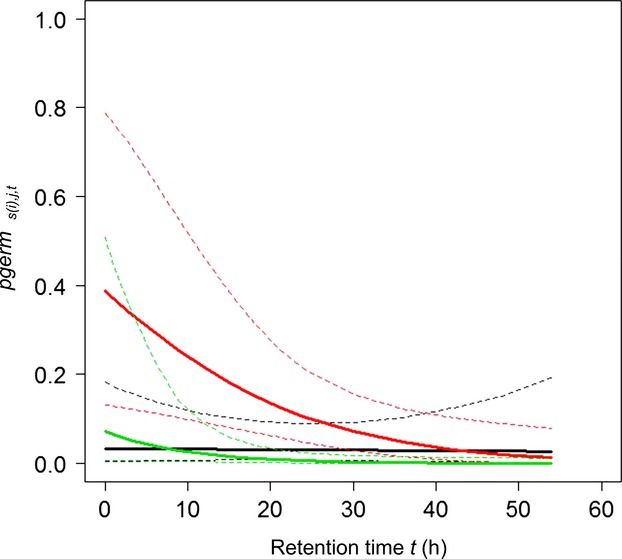
Germination probability. Variation of germination probability (*pgerm*_*s(i),j,t*_) with seed retention time, in red deer, for *Calluna vulgaris* (in black), *Juncus effusus* (in red), and *Plantago media* (in green). Plant–animal pairs that were misestimated due to low sample size are excluded. Bold and dashed curves, respectively, represent the median and its 95% credibility interval.

In controls, the germination percentage of noningested seeds of *Plantago media* (91 ± 2%, *n* = 4 × 100), *Prunella vulgaris* (75 ± 2%), and *Trifolium pratense* (70 ± 6%) were all higher than 70%. Germination was lower for *Juncus effusus* (23 ± 3%) and *Calluna vulgaris* (8 ± 2%), while *Rubus fruticosus* did not germinate at all. Considering all the fecal samples, all the six plant species germinated at least once. Per 100 g of feces, 26.94 (±36.11) seedlings germinated from red deer, whereas only 5.40 (±13.34) and 4.71 (±9.86) germinated, respectively, from wild boar and roe deer. All vectors taken together, we observed seedlings in only 53 of 154 feces. In roe deer, isolated seedlings of *Calluna vulgaris*, *Plantago media,* and *Prunella vulgaris* germinated in 10 feces. In red deer, we observed up to nine seedlings of *Juncus effusus* in all but one feces; up to four seedlings of *Calluna vulgaris* per feces, in 11 feces; up to two seedlings of *Plantago media* per feces, in seven feces; and up to two seedlings of *Trifolium pratense* per feces, in three feces. In wild boar, only *Plantago media* and *Rubus fruticosus* germinated in small numbers (respectively, up to two seedlings per feces, in two feces; and up to four seedlings per feces, in four feces), while *Rubus fruticosus* did not germinate at all in controls. As a consequence of the rarity of germination events, median germination probabilities were misestimated to either zero or 1, except for *Calluna vulgaris* (maximal *pgerm*_*s(i),j,t*_: 0.058 [0.013; 0.243]), *Juncus effusus* (0.388 [0.136; 0.787]), and *Plantago media* (0.071 [0.001; 0.508]) in red deer. For these three plant–animal pairs, germination probabilities decreased rapidly with retention time (Fig.5).

## Discussion

We showed that seed retention time varied among plant–animal species pairs chosen for their contrasting morphological and physiological traits and that germination probability was low and tended to decrease with retention time. Animal and seed characteristics probably interact through the endozoochorous process, with consequences on the spatial structure of the seed shadow as well as the resulting plant distribution and community patterns.

Our model is the first to quantify jointly retention time and postdispersal germination probability in an explicit dynamic framework. Our experiment also represents the first attempt to estimate seed retention time by a small browser ruminant species (roe deer) and a relatively small hindgut fermenter (wild boar). So doing, we provide experimental-based data suitable to calibrate mechanistic models of zoochorous plant dispersal similar to those of Will and Tackenberg (2008) or D’hondt et al. (2012). These innovations come with some limitations imposed by the usual constraints of experimental monitoring of captive animals of wild origin, the first one being small sample size. The limited availability of captive ungulates in experimental platforms and the difficulty of controlling animal behavior during the experiments are particularly restrictive when studying wild species. Nevertheless, our sample size (six replicates per animal species) was higher than in previous studies (four replicates for fallow deer or moose, and five for rabbit, cattle, sheep, donkey, and horse, by Cosyns et al. 2005; Mouissie et al. 2005; Seefeldt et al. 2010). Furthermore, the small size of the plant species assemblage and the limited number of animal individuals limited the extent of interspecific variability in seed retention time, thus making it difficult to disentangle the effects of vector and plant traits in a quantitative way. In the prospect of improving the empirical validation of these models, we strongly encourage the replication of our data to fit our model over longer study periods and with larger animal and plant species samples.

### Seed retention times

Our seed retention times are consistent with those obtained for particles or food in previous experiments (Holand 1994; Jiang and Hudson 1996; Behrend et al. 2004; Elston and Hewitt 2010). Yet, contrary to our initial hypotheses, differences in feeding preferences and body mass did not translate into shorter retention times in roe deer than in red deer. Wild boar exhibited longer retention times (>36 h) than the two ruminant species for all the plant species concerned, contrary to what we expected following Illius and Gordon’s equations. Separating the effects of body mass from those of digestion strategy would require intraspecific replication (in red deer, for example, because of its high sexual dimorphism and large range of body masses among individuals). In our case, this was not feasible due to the difficulty of finding adequate wild animals available for experimentation. However, our results are unlikely to be solely attributable to differences in body mass as the mass difference between wild boar and red deer equaled that between red deer and roe deer. Additionally, body mass appeared unrelated to retention time in previous studies (Schwarm et al. 2008; Steuer et al. 2011), which suggests that the effects of digestion strategy may dominate those of body mass on seed retention time.

Our results showed that seeds may be sorted according to their size and shape, as was previously shown for other digested particles (Clauss et al. 2009). As predicted, small and light seeds (*Juncus effusus* and *Calluna vulgaris*) and round seeds (*Calluna vulgaris* and *Trifolium pratense*) were excreted faster than other seeds by the two ruminant species. In wild boar, small and round seeds seemed to be excreted later than others, suggesting that particles are sorted through different processes in nonruminants compared to ruminants. Hence, animal and seed characteristics interact and influence seed retention time. We further noticed that the differentiation between seeds was more marked in red deer, which exhibited a wider range of retention times (from 3 h to 36 h: *Trifolium pratense* and *Calluna vulgaris* first, followed by *Juncus effusus* and *Plantago media*, and then *Prunella vulgaris* and *Rubus fruticosus*) than roe deer (from 18 h to 36 h). Clauss et al. (2009) also found that browsers tend to stratify the gut content less than mixed feeders and grazers, suggesting that red deer may increase heterogeneity in dispersal distances among plant species, which could increase spatial heterogeneity among plant communities, while roe deer would rather increase local heterogeneity and spatial homogeneity. Hence, each vector is likely to affect plant community at different spatial scales.

The GPS monitoring of wild animals revealed that red deer covered longer distances in a straight line (2.6 km in average, and up to 3.5 km) than wild boar (2.2 km and up to 3.1 km) than roe deer (1.7 km and up to 2.0 km) in 48 h (unpublished data). Moreover, Adrados (2002) showed that a male red deer can cover up to 10.3 km in 24 h between two seasonal parts of its annual home range, and female roe deer can cover up to 1.4 km in 6 h during the rut (Richard et al. 2008). The distances covered by the three animal species are longer than one kilometer, which correspond to long dispersal distances (Cain et al. 1998; Higgins and Richardson 1999). Thus, the three ungulates would induce long-distance dispersal rather than short-distance dispersal, suggesting that they could impact connectivity among distant populations and colonization processes.

### Seed survival

As we expected, germination probability decreased with retention time in all plant–animal pairs. We were only able to estimate germination probability for three plant–animal pairs. As all the six plant species germinated either in feces or controls, low germination probabilities unlikely result from the controlled conditions in the germination chamber. Hence, the main possible experimental limitation that could have contributed to our results is the limited time allowed to the germination tests, which could have been too short for some dormant seeds. Indeed, some studies have shown that a considerable number of seeds migrate from decomposing feces to deeper soil layers instead of germinating (Malo and Suárez 1995a,b; Pakeman et al. 1999; Jaroszewicz 2013). We therefore suggest replicating our germination data allowing for longer experimental durations and conditions closer to the natural context to strengthen our conclusions.

Although wild boar excreted more seeds than the other two species, fewer seeds germinated in its feces. Hence, longer retention time and the resulting long dispersal distances associated with this vector come at the cost of the mortality of most seeds. Contrastingly, the fact that fruit seeds (*Rubus fruticosus*) only germinated in wild boar feces (and not in controls) suggests that endozoochory may not systematically be costly to seeds and can even favor germination. *Rubus* seeds are enclosed by a hard endocarp that impedes water imbibition (Wada et al. 2011), which increases seed survival but impairs germination in the absence of aggressive physical conditions such as digestive enzymes. Our results therefore suggest that slow-digesting animals are more effective to disperse resistant seeds over longer distances than ruminants.

## Conclusion

Our study focused on an individual process limited in space and time. However, we have to keep in mind that endozoochory is a spatially and temporally continuous process, involving a wider range of plants consumed at different times in different places, as long as the animal is feeding. Endozoochory by large ungulates may allow long-distance seed dispersal, favoring connectivity among distant populations within metapopulations or the colonization of unoccupied suitable patches. Our experimental findings and the associated modeling framework have key implications for understanding the role of zoochorous dispersal in shaping plant communities. Different vectors are likely to impact the dynamics of plant species at different spatial scales, not only according to their daily movements within their home ranges, but also according to their life history traits and those of the dispersed plants. Therefore, spatial patterns of plant distributions and community composition are likely to depend in a predictable way on the composition and relative abundance of herbivores, not only through grazing, but also as connectivity agents. Comparing the relative influence of endozoochory to other plant dispersal modes will require extensive experimentation combining animal displacement tracking and seed retention-time estimations similar to ours. Our results support a more extensive assessment of the influence of wild ungulates as vectors of plant dispersal and their impact on spatial patterns of community composition and connectivity.

## Data accessibility

Raw data available from DRYAD: entry doi: 10.5061/dryad.14vt7.

## Supporting Information

**Appendix S1.** Description and location of experimental platforms.

**Appendix S2.** Posterior predictive checking.
