# Supplementary material for: Temporal dynamics of seed excretion by wild ungulates: implications for plant dispersal
Source: Ecol Evol. 2015 Jun 6;5(13):2621–32. doi: 10.1002/ece3.1512 (PMC4523358; doi:10.1002/ece3.1512)
Supplement: Supplementary file 1 [file ece30005-2621-sd1.docx]

**Appendix S1 – Description and location of experimental platforms.**

All experiments were conducted in agreement with the ethical standards of animal manipulation as defined by the French laws on animal welfare (see Décret n° 2013-118), in three platforms with relevant licenses for the capture, care, and use of nonprotected wild animal species (see the licenses numbers, issued by the French Government, hereafter). These permits also cover our study. Indeed, additional ethical approval was not required for our kind of nonintrusive experimental protocol (without injection). Animals were used directly on site, in each of the three platforms, under the supervision of veterinarians who also contributed to the experimental design (within a partnership: animal and staff rental). All efforts were made to minimize animal stress due to confinement and human proximity, especially by choosing the most impregnated animals. Enclosures and boxes were from 10 to 15 m², side by side, and separated by an open wall, so that each animal could see its congeners. Animals in enclosures had free access to a covered area. Boxes were illuminated by windows. During the experiments, the animals had free access to freshwater and received their usual food every day. After the experiments, animals were released within their usual enclosure.

Décret n° 2013-118 du 1er février 2013 relatif à la protection des animaux utilisés à des fins scientifiques. Article 1, Section 6:

- Sous-section 1, Article R. 214-88, 7° ;

- Sous-section 2, Paragraphe 2 ;

- Sous-section 3, Paragraphe 1 and 2.

<http://www.legifrance.gouv.fr/affichTexte.do?cidTexte=JORFTEXT000027037840&dateTexte=&categorieLien=id>

- *Roe deer*

Roe deer were provided by the I.N.R.A (French National Institute for Agricultural Research) “Behaviour and Ecology of Wildlife” unit (C.E.F.S). I.N.R.A is a public research institution under the joint authority of the French Ministry of Higher Education and Research and the French Ministry of Agriculture, Food Industry, and Forests. C.E.F.S unit works on natural and captive populations of ungulates and possesses an experimental enclosure of about 20 hectares, comprising 7 subenclosures (from 0.5 to 1 hectare), with 18 roe deer (14 females and 4 males), in Gardouch (see location in the figure hereafter).

C.E.F.S website: <http://www6.toulouse.inra.fr/cefs/>

C.E.F.S has relevant authorizations for the care and use of roe deer.

*Name:* I.N.R.A’s experimental platform 0035 in Gardouch

*Approval number:* A-31-210-001, by the French Government

*Date of issue:* 21 July 2011

*Person in charge:* Michèle Marin, president of the I.N.R.A regional center of Toulouse.

*E-mail:* [Michele.Marin@toulouse.inra.fr](mailto:Michele.Marin@toulouse.inra.fr)

*Address*: I.N.R.A – C.E.F.S, Chemin de Borde Rouge, Auzeville, BP 52627, 31326 Castanet-Tolosan Cedex, France.

*On-site delegate:* Hélène Verheyden, C.E.F.S unit manager

*E-mail:* [Helene.Verheyden@toulouse.inra.fr](mailto:Helene.Verheyden@toulouse.inra.fr)

- Red deer

Red deer were provided by the Alfort Veterinary School (E.N.V.A), a higher education and research establishment under the authority of the French Ministry of Agriculture, Food Industry, and Forest. E.N.V.A possesses a field station of about 100 hectares of agricultural land and an experimental deer unit, with 40 red deer (all females but 2 males), in Champignelles (see location in the figure hereafter).

E.N.V.A website: <http://www.vet-alfort.fr/web/en/291-champignelles.php>

E.N.V.A has relevant authorizations for the use of deer.

*Name:* Deer program

*Approval number:* 89129, by the French Government

*Type:* B category, meat and reproduction

*Person in charge:* Hélène Benoît

*E-mail:* [hbenoit@vet-alfort.fr](mailto:hbenoit@vet-alfort.fr)

*Address:* Centre d’application de l’E.N.V.A, domaine de Croisil, 89350 Champignelles, France.

- *Wild boar*

Wild boars were provided by Animal Contact studio, specialized in animal training for cinema, television, publicity, photography, and events, in Ladon, France (see location in the figure hereafter). Animal Contact breeds a wide range of animals, both domestic and wild. The studio possesses a female and a male wild boar. The Animal Contact team is composed of one specialized trainer per species and a veterinarian.

Animal Contact website: <http://www.animal-contact.com/>

Animal Contact possesses certificates of competence for the use and detention of nondomesticated animals.

*Certificate number:* **45-10-008, 45-12-008** and **45-12-009**, by the French Government

*Person in charge:* Muriel Bec

*E-mail:* [info@animal-contact.com](mailto:info@animal-contact.com)

*Address:* Animal Contact, 47 Rue Jean-Martin Chambon, 45270 Ladon, France.


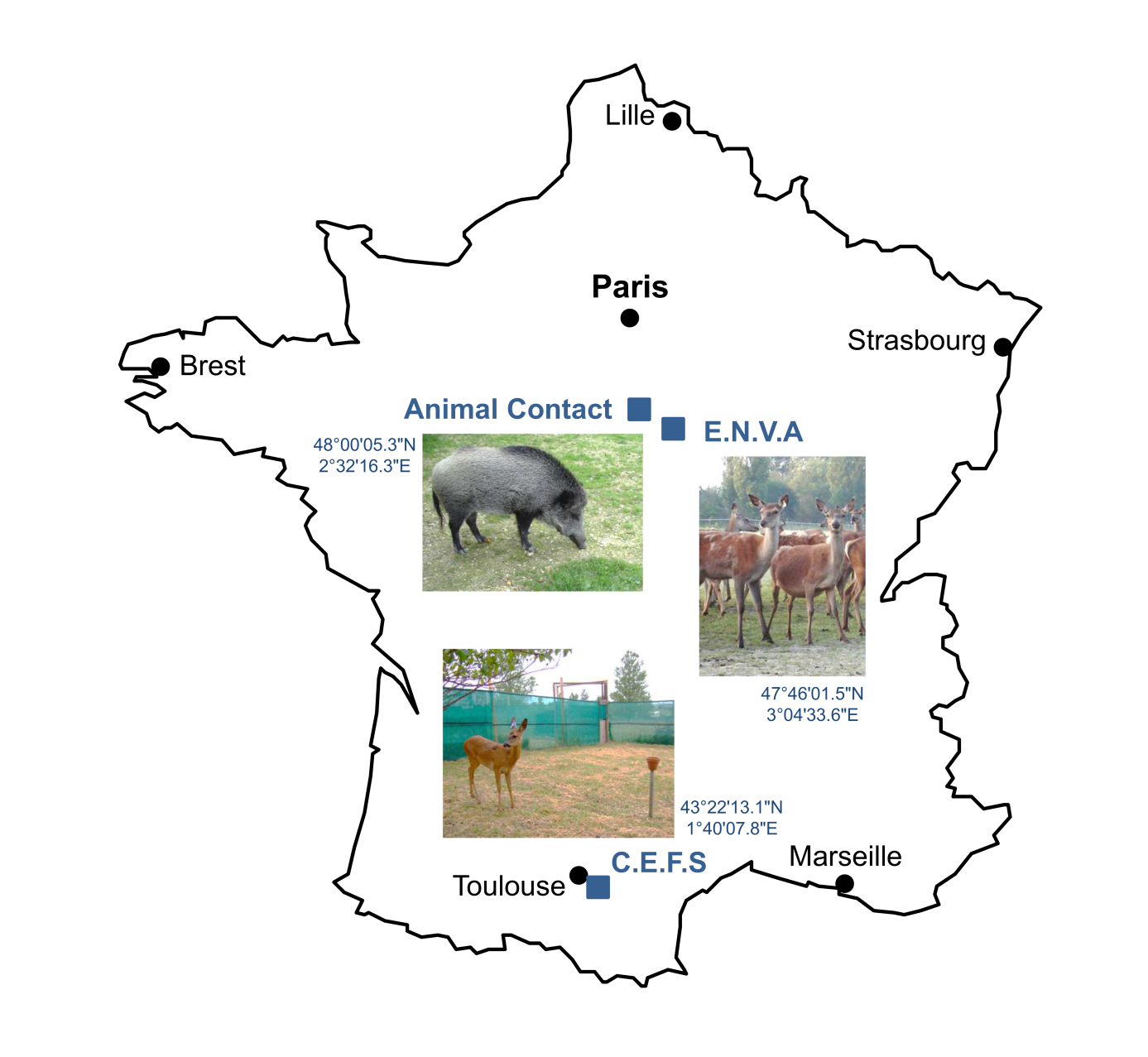


**Figure:** Map of France showing main French cities (black circles), the three study sites (blue squares): Ladon (Animal Contact, for wild boar), Champignelles (E.N.V.A, for red deer) and Gardouch (C.E.F.S, for roe deer), and their GPS coordinates (in Degrees, Minutes, and Seconds).
