# Supplementary material for: Temporal dynamics of seed excretion by wild ungulates: implications for plant dispersal
Source: Ecol Evol. 2015 Jun 6;5(13):2621–32. doi: 10.1002/ece3.1512 (PMC4523358; doi:10.1002/ece3.1512)

**Appendix S2 – Posterior predictive checking.**

**(A)** Correlation between *Y.rep_i,j,t_* (replicated seed counts) and *Y_i,j,t_* (observed seed counts in the dissected sample). **(B)** Correlation between *G.rep_i,j,t_* (replicated seedling counts) and *G_i,j,t_* (observed seedling counts in the germination sample). A perfect fit corresponds to the 1-1 line (in blue).


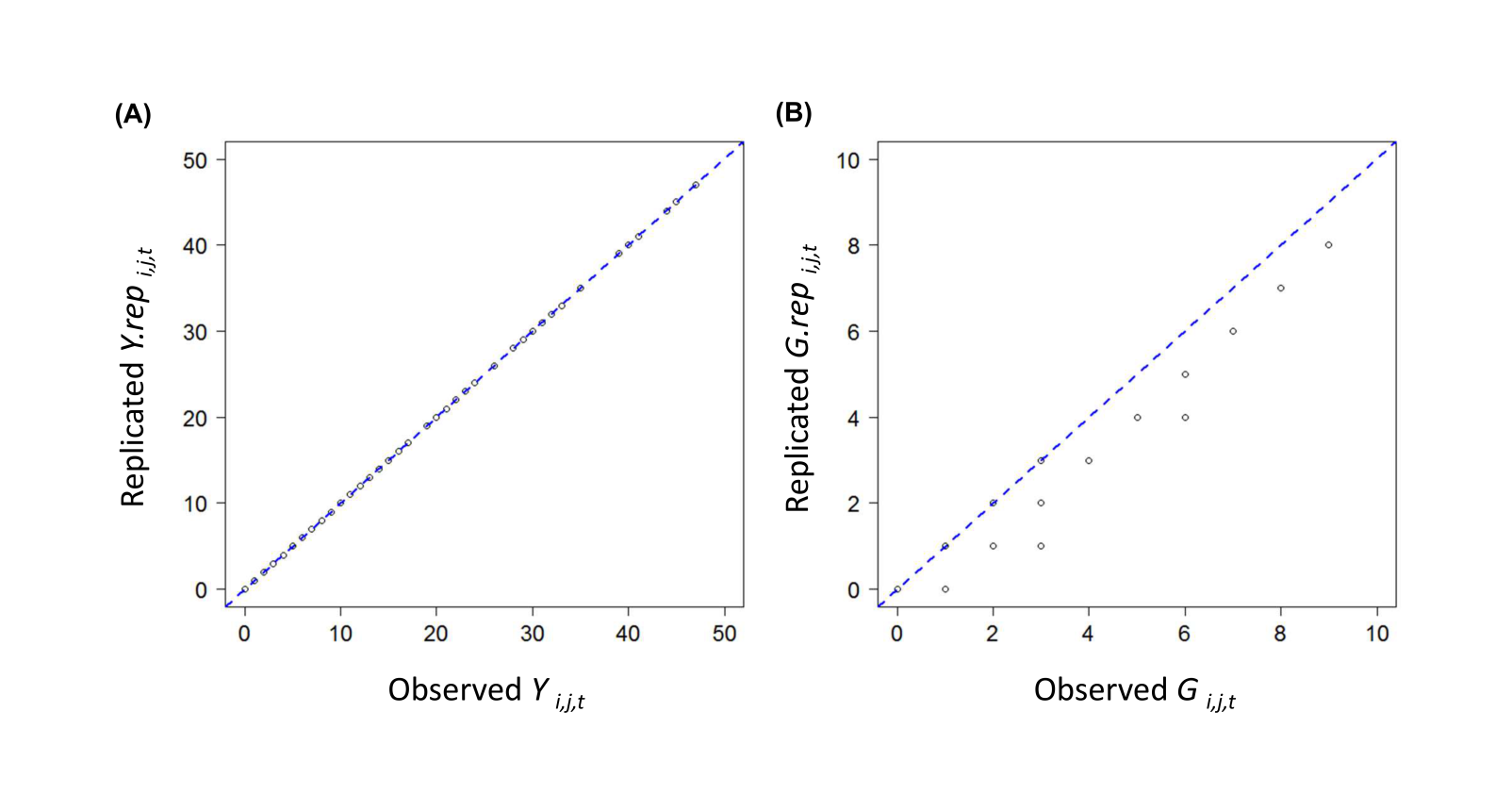

Supplement: Supplementary file 2 [file ece30005-2621-sd2.docx]
